# Rethinking immigration policy theory beyond ‘Western liberal democracies’

**DOI:** 10.1186/s40878-018-0071-9

**Published:** 2018-03-01

**Authors:** Katharina Natter

**Affiliations:** Department of Sociology, University of Amsterdam, Nieuwe Achtergracht 166, 1018 WV Amsterdam, The Netherlands

**Keywords:** Immigration policy theory, Migration policy, Political regimes, Policy-making, Democracy, Autocracy, Morocco, Tunisia

## Abstract

How do political systems shape immigration policy-making? Explicitly or implicitly, comparative politics and migration policy theories suggest a ‘regime effect’ that links specific dynamics of immigration policy to liberal democracy. The literature’s dominant focus on so-called ‘Western liberal democracies’, however, has left the ‘regime effect’ largely untested and research on variations and similarities in immigration policymaking across political systems strikingly undertheorized. This paper challenges the theoretical usefulness of essentialist, dichotomous categories such as Western/non-Western or democratic/autocratic and calls for a more nuanced theorizing of immigration policy-making. It proposes a two-dimensional classification of immigration policy theories, distinguishing between ‘issue-specific’ theories that capture immigration policy processes regardless of the political system in place and ‘regime-specific’ theories whose insights are tied to the characteristics of a political system. The paper also advances the ‘illiberal paradox’ hypothesis to explain why illiberal, autocratic states may enact liberal immigration policies. This theoretical expansion beyond the ‘Western’ and ‘liberal’ bubble is illustrated by an analysis of immigration policy-making in 21st century Morocco and Tunisia. Showing how domestic and international institutions, interests, and ideas shape immigration policy-making in Morocco’s monarchy and Tunisia’s democratic transition, the paper investigates the broader role of political systems in immigration politics and herewith seeks to contribute to a more general and global theorization of immigration policies.

## Introduction

### Setting the scene

Over the past three decades, research on immigration policy-making has flourished. To explain why so-called ‘Western liberal democracies’ have liberalized immigration despite popular demands for restriction, scholars have pointed at the democratic character of these states. Freeman (1995) argued that immigration policy-making in democracies follows the pattern of ‘client politics’ because the costs of immigration are diffused among the entire electorate, while benefits are concentrated within a small pool of entrepreneurs. Hollifield (1992a, 1992b), in turn, showed how the political logic of democratic nation-states pushes towards restrictiveness, while the economic logic of global market liberalization pushes for openness towards immigration. This ‘liberal paradox’ or ‘embedded liberalism’ would explain why politicians’ discourses about immigration tend to be more restrictive than implemented policies. Similarly, Sassen (1996) and Joppke (1998) argued that the rise of an international human rights regime and the activism of national courts, characteristic of liberal democracies, have limited the power of states to curtail migrants’ rights.

These theoretical insights have systematized our ways of thinking about immigration policy. Yet, their almost exclusive focus on ‘Western liberal democracies’ is problematic in three regards. First, the term ‘Western liberal democracy’ is rarely (if ever) explicitly defined and theorized by the authors. It is used as a shortcut to signal that these theories mainly apply to an ‘exclusive club’ of countries - essentially North and Western Europe, North America, Australia and New Zealand, but more recently also Southern and Eastern Europe and sometimes even Japan - without providing a substantive explanation for this limitation. Second, by linking democracy and liberal immigration policy, these scholars have implicitly suggested a ‘regime effect’. The hypothesized effect of democracy (or, in fact, authoritarianism) on immigration policy-making has, however, not been tested thoroughly so far. This is due to a lack of empirical research on immigration policy-making in a variety of political systems, as well as the assumption that authoritarian policy-making requires a different set of theories, herewith overlooking the possibility that current theories might also apply to more ‘autocratic’ states. Finally, this exclusive focus has narrowed academic insights into the broader role of states in international migration, and limited policy-makers around the world in their attempt to develop more effective approaches towards migration.

This paper rethinks immigration policy theories by moving beyond the dominant approach that links theories of immigration policy to a country’s preconceived--and potentially reductionist--categorization as ‘Western’ or ‘non-Western’, ‘democratic’ or ‘autocratic’. Rather than focusing on binary regime types, this paper looks at the structure, functioning and practices of a country’s political system and asks: *How do political systems shape immigration policy-making?* It proposes a two-fold classification of immigration policy theories, distinguishing between features of immigration policy-making that are intrinsic to the issue of immigration and thus valid regardless of the political system in place, as well as those that indeed seem to portray a ‘regime effect’. The paper also advances the ‘illiberal paradox’ hypothesis according to which illiberal, autocratic regimes can more easily adopt open immigration policies if these fit their priorities, as they are more autonomous from popular demands for closure compared to liberal-democratic regimes.

These theoretical reflections have emerged from empirical research conducted for my Ph.D. on immigration policy-making in twenty-first century Morocco and Tunisia, two countries that challenge conventional regime classifications and their expected effects on immigration policy. While Morocco’s monarchy consolidated its power since the 2000s through limited constitutional reforms, Tunisia experienced a radical break in its political system in 2011, passing from an authoritarian one-party regime to the (ongoing, albeit staggering) establishment of democracy (Hinnebusch, 2015; Vermeren, 2009; Willis, 2012). Over the same period, both countries have also started to craft their immigration policy, with Morocco showcasing a fundamental policy shift in 2013 and Tunisia portraying a striking continuity in its immigration policy throughout the revolution. The within- and across-country comparative case studies on Morocco and Tunisia thus provide critical insights on immigration policy-making in the wake of diverging political transformations and beyond the usual scope of immigration policy theories.

The paper first reviews main immigration policy theories. It then outlines the conceptual stakes of this article by challenging dominant categorizations in migration research and reviewing the emerging literature to which this paper seeks to contribute. Second, the article dissects policy-making in Morocco and Tunisia. Based on collected primary sources and 110 semi-structured interviews conducted with key informants within Moroccan and Tunisian civil society and state institutions between October 2016 and May 2017, it analyzes the emergence of immigration as a ‘public problem’ in Morocco and Tunisia, and the processes underlying immigration policy changes or continuities since the 2000s. Finally, the paper confronts the empirical material with existing immigration policy theories to explore the role of political systems on immigration policy-making. The suggested categorization of regime-specific and issue-specific features of immigration policy and the ‘illiberal paradox’ hypothesis seek to provide food for thought for a more global theorization of immigration policy.

### A quick overview of immigration policy theories

Existing reviews of immigration policy theories (see: Bonjour, 2011; Boswell, 2007; Castles, 2004; Hollifield, 1992b; Massey, 1999; Meyers, 2000) suggest four primary determinants of immigration policy: (1) the role of socioeconomic interests at the domestic level, operating via interest groups and public opinion; (2) the importance of foreign policy and diplomatic interests; (3) the role of state institutions’ potentially conflicting interests; and (4) the impact of international norms on national policy-making. Explanations of immigration policy vary along two main dimensions: the *factors of analysis* considered, namely the emphasis on the role of ideas, interests or institutions in immigration policy-making; and the *level of analysis* adopted, namely the localization of the primary source of immigration policy within society, the state or the international arena. Table 1 provides an overview of these theories and their primary foci.

**Table 1:**
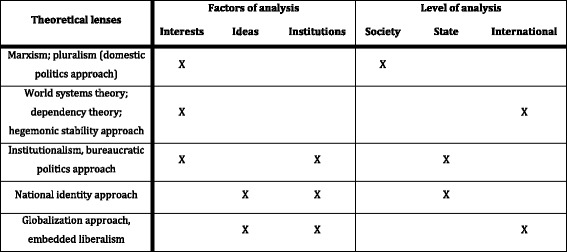
Categorizing immigration policy-making theories

Classical political economy approaches analyze different actors’ interests (costs and benefits) to determine which problems are set on the agenda and how decisions are taken. This approach locates the origin of immigration policy within society, and tends to reduce the state to a neutral arena captured by economic and societal interests. Within this tradition, Marxist approaches see immigration policy as a result of capitalist interests, as liberal policies towards low-skilled workers and irregular migrants are in the interests of employers, who are keen to increase the pool of dependent and vulnerable workers (Castles & Kosack, 1985). Pluralist or ‘domestic politics’ approaches suggest that societal interests do not run along classes, but along political parties, interest groups, or client networks. Most prominently, Freeman (1995, p. 2006) theorized that immigration policy-making in liberal democracies tends to be characterized by client politics because the costs of immigration are diffused and its benefits concentrated. International relations theories lift these approaches to the international realm: While world systems and dependency theories within the Marxist tradition posit that immigration policies reflect the global market structure (Portes & Walton, 1981), hegemonic stability approaches within the realist tradition postulate that immigration policies reflect the interests of geopolitically dominant states. These theories are particularly relevant to understand refugee policies or the use of migration as a foreign policy tool, leading to a ‘diplomacy of migrations’ by origin or destination countries (Mitchell, 1989; Teitelbaum, 1984).

In contrast to political economy analyses, institutionalist explanations are closely tied to a political sociology of the state. Institutionalism grants the state (partial) autonomy from societal interests and focuses on inter-institutional dynamics and the fragmentation of state interests. Bureaucratic politics approaches highlight the role of turf wars - disputes between institutions over spheres of influence - in immigration policy-making, and of path dependency dynamics created by previous policy decisions. Joppke (1998) for instance emphasized the importance of lawyers and judges in enshrining migrants’ rights against other state institutions’ attempts to curtail them (such as Ministries of Interior); and Hansen (2002) explained liberal policies towards colonial migrants in France and the UK, as well as towards asylum seekers in Germany through path dependence dynamics.

A third set of theories emphasizes the historical context of immigration policy-making and focuses on the structural effects of ideas and institutions in constraining policy-makers. For instance, the national identity approach has been widely used to show how immigration policies tie into a country’s particular national history, identity or political institutions. In this vein, Zolberg (1978) has analyzed how, in Western Europe and North America, changing national migration policies reflected the emergence of modern states, the international system, and capitalism between the 16th and the 20th centuries. Later, Brubaker (1992) traced French and German immigration policies back to these countries’ distinct histories of nationhood. Research inspired by globalization theories shifts these dynamics onto the international level and focuses on how global norms and institutions shape national immigration policy-making. Most prominently, Sassen (1996) argued that the rise of an international human rights regime constrains nation states, safeguarding migrants’ rights against national administrations’ attempts to curtail them. Similarly, Hollifield (1992b) argued that a dominant liberal ideology seeking to protect individual rights and to globalize national market economies limits nation states in their immigration policy-making autonomy.

As none of these theories can claim to provide a freestanding account of immigration policy, most studies combine different theoretical insights to explain observed policy processes. For instance, Timmer and Williams (1998) use quantitative analyses and draw on Marxist and globalization theories to distill the economic factors and international policy diffusion mechanisms that led to immigration restrictions in Argentina, Australia, Brazil, Canada and the US before the 1930s; and Calavita (1992) joins a bureaucratic politics analysis with a pluralist approach in her study of the US Bracero Program through the lens of the Immigration and Naturalization Service. Ultimately, most immigration policies - regardless of the political system in place - are likely determined by the dialectic between interests, institutions, and ideas evolving at the intersection of domestic and international spheres. The crux is to specify the dynamics between factors and the relative weight of each of them.

### Going beyond binary world (di)visions in migration policy research

Due to the dominant research focus on ‘Western liberal democracies’, most countries in the world are not covered by existing theories – including some with the highest levels of immigration or emigration, such as the Gulf countries, Malaysia, Indonesia or Côte d’Ivoire. This is due to two interrelated assumptions that are widespread among migration researchers and policy-makers. First, there is a tendency to split the world into migrant destinations situated in the ‘Global North’ and migrant origins situated in the ‘Global South’. This ignores that nearly 50 % of international migrants – trend rising – live in countries categorized as ‘southern’ (UNPD, 2013, p. 1). Second, this categorization along development levels or political geographies frequently overlaps with assumptions about political regimes: Destinations in the ‘Global North’ are often implicitly cast as liberal democracies, while origins in the ‘Global South’ are classified as autocracies or, at best, malfunctioning democracies. This disregards not only the existence of autocracies as main migrant destinations - such as the Gulf countries -, but also the autocratic histories of countries such as Spain and Greece, and the existence of established democracies in Asia and Latin America, such as India and Brazil.

This does not mean that migration policies outside the ‘Western liberal-democratic’ sphere have not been subject to research at all. Yet, most studies focus on emigration and diaspora policies (de Haas & Vezzoli, 2011; FitzGerald, 2006; Gamlen, 2008; Miller & Peters, 2014), or look at policy-making in the context of ‘externalization’ (Adepoju, van Noorloos, & Zoomers, 2010; Lavenex & Uçarer, 2002). Where studies on immigration policy in such countries exist - be they qualitative (González-Murphy & Koslowski, 2011; Paoletti, 2011; Poutignat & Streiff-Fénart, 2010) or quantitative (Breunig, Cao, & Luedtke, 2012; Shin, 2017) - they often treat states as single, homogeneous entities without paying attention to the fragmentation of state interests, and ignore their decision-making autonomy in front of international actors. Structural explanations of how such emigration, diaspora and immigration policies are formed, and what different interests have to be negotiated in the process are thus largely missing.

Only recently, researchers started to question the assumptions of mainstream immigration policy theories. In the context of the Americas, FitzGerald and Cook-Martín (2014) challenged Western-centric preconceptions about the link between liberal immigration policy and democracy by showing that North American democracies were the first to establish ethnic immigration selection criteria and the last to abolish them, long after most Latin American autocracies did so. Acosta Arcarazo and Freier (2015), in turn, questioned Hollifield’s liberal paradox hypothesis by analyzing the ‘populist liberalism’ in several Latin America democracies, where political discourses on immigration have overall been more liberal than implemented policies. Working on the Gulf countries, Thiollet (2016) investigated the existence of an ‘illiberal transnationalism’ on immigration in the region; and Norman (2016a, 2016b) hypothesized that countries such as Morocco, Turkey, and Egypt are consciously pursuing a ‘policy of ambivalence’ towards migrants, preferring to grant migrants rights through ad-hoc policy decisions rather than through legal changes, herewith leaving open the option of future, rapid backlashes.

This paper builds on these recent empirical and theoretical insights that question the assumed link between liberal democracies and immigration policy-making and seeks to go one step further by overcoming the dichotomous and - as I hope to show - ultimately overrated democracy/autocracy categorization in migration policy research. In fact, autocratic and democratic features can be found in most political systems around the world. Civil society organizations (CSOs) can play a fundamental role in more autocratic political systems (Keck & Sikkink, 1992; Natter, 2014b; Russell, 1989), while some policy processes in democracies are, in fact, free from much democratic control (such as the signing of executive orders or presidential decrees). Instead of categorizing countries as either democratic or autocratic, a more fruitful approach is thus to look for autocratic and democratic policy processes and practices within each political system (Glasius, 2017). Confronted with the variety of real-world political systems, political science research has long abandoned the democracy/autocracy dichotomy and replaced it by an understanding of political systems along a spectrum, leading to the proliferation of new in-between terms such as limited democracies, illiberal democracies, liberal autocracies, or competitive authoritarianism (see: Brooker, 2014; Diamond, 2002; Linz, 2000). This article seeks to integrate these nuances into the migration policy literature that still overwhelmingly operates with dichotomous regime classifications.

More importantly, however, the more or less democratic character of a state is just one of its defining features. Reducing the discussion of political systems to the democracy/autocracy dichotomy disregards many other key features of a state that determine policy processes, such as the state’s organization into ministries and administration, the role of lobbies and voters, or the institutionalized interactions with courts, the media, CSOs and international organizations. As highlighted by Tilly (1992), social sciences tend to focus on the differences in state formation while disregarding the fundamental similarities in the nature of modern statehood around the world. Because political regimes rarely match the ideal-types of democracy and autocracy, looking for ideal-typical democratic or autocratic immigration policy processes is a fundamentally flawed exercise. By looking at the structure, functioning and practices of Morocco’s and Tunisia’s political systems for the subsequent analysis of their immigration regimes, I hope to overcome some of the limitations of earlier, regime-focused analyses.

## Immigration policy and political transformations in Morocco and Tunisia

### A double paradox

Morocco and Tunisia are two theoretically critical cases to study immigration regimes beyond the usual scope of immigration policy theories. Both countries are quintessential ‘emigration states’ (see Gamlen, 2008) with extensive ‘migration cultures’, whose authorities have concentrated their policy efforts throughout the 20th century on encouraging and channeling emigration, as well as controlling or courting their diasporas (Brand, 2002; de Haas, 2007; Natter, 2014a). This exclusive focus on emigration has waned, with immigration gaining increasing political salience, particularly in Morocco. This has been partly due to the political framing of Morocco and Tunisia as ‘transit countries’(see Düvell, 2012 for a critical discussion of the ‘transit migration’ concept). Currently, a second reframing process is taking place, in which Morocco and Tunisia are increasingly portrayed and also portray themselves as immigration countries. This has led to new public policies, but also to increased civil society activism and scientific interest in the topic.

The growing political salience of immigration is, however, only partly rooted in changes on the ground: In Morocco, immigration from Africa and Europe has always existed in the context of education, pilgrimage, trade, or war. Since sub-Saharan migrants started to join Moroccan migration towards Europe in the mid-1990s, immigration to Morocco has also increased and diversified, even if overall numbers remain small. Census data show moderately rising immigrant numbers - from 50,200 to 86,200 between 2004 and 2014, representing respectively 0.17 and 0.25% of the Moroccan population (HCP, 2009, 2014). Census data certainly underestimates Morocco’s total migrant population, but even higher estimates of around 200,000 migrants do not challenge the overall conclusion that immigration remains a minor phenomenon in Morocco - especially when considering the size and continuous growth of Morocco’s emigrant population, estimated at 4 million in 2012 (Berriane, de Haas, & Natter, 2015).

In Tunisia, European immigration has been a constant in the country’s post-independence history, and immigration from sub-Saharan Africa started to grow in the 2000s with the expansion of the private university sector and the relocation of the African Development Bank (BAD) from Abidjan to Tunis between 2003 and 2014. Also here, census data show that immigrant numbers remain modest, increasing from 35,200 to 53,500 between 2004 and 2014, i.e., from 0.35 to 0.49% of the population (INS, 2015). However, census data does not capture the unprecedented immigration from Libya since 2011, given that most Libyans remain in Tunisia on a tourist visa. With estimates ranging from anything between 8000 to 800,000 Libyans in Tunisia, this development shows Tunisia’s - at least temporary - transformation into a destination country.

Politically, Morocco and Tunisia followed strikingly different paths. In Morocco, political power remains concentrated within the ‘Makhzen’, a network of politicians, families, and businessman centered around the King. Despite the considerable continuity of authoritarian power structures, punctual liberalizations since the 1990s have extended political, media, and women’s rights, and CSOs have slowly grown into important actors on certain policy issues. In 2011, popular protests led to a new constitution that granted more (although still limited) power to the parliament and government and enshrined new civil and political rights for Moroccans and foreigners (Boukhars, 2011; Cubertafond, 2001; Vermeren, 2009).

In contrast to this continuity in political life, Tunisia’s 2011 ‘Jasmin revolution’ broke with the authoritarian regime of Ben Ali where power was concentrated in presidential hands, the state party was instrumentalized, and freedom of speech and civil society activism openly repressed. The revolution in 2011 showcased the glaring contradictions between Tunisia’s social reality of deep economic inequalities and widespread corruption, and its political image abroad as an economically prosperous, secular, and progressive country. Several years later, while the political democratization process has been relatively successful, the long-lasting economic downturn and the ongoing security issues, particularly in the South of Tunisia, continue to challenge the political transition (Beau & Tuquoi, 2011; Camau & Geisser, 2003; Hibou, 2006).

The analysis of Morocco’s and Tunisia’s political transformations and immigration regimes over the last two decades shows that the two countries have reacted differently to changing migratory realities. This gives rise to a double paradox: Tunisia has seen an important increase in immigration, but policies have remained stable. In contrast, Morocco set immigration on the political agenda and enacted fundamental policy changes despite only moderate growth in immigration. This is particularly striking considering the continuity in Moroccan political life compared to the regime change that Tunisia has experienced since 2011. These observations point to a disconnection between (1) the magnitude of immigration as a phenomenon, (2) its political treatment and the public policies developed to address it, and (3) the political, contextual developments within which migration and migration policy occur. Table 2 summarizes this double paradox.

**Table 2:** A dual paradox in Moroccan and Tunisian immigration policy-making

	Morocco	Tunisia
Magnitude of immigration	Low	High
Politicization of immigration	High	Low
Magnitude of political change	Low	High

How can we make sense of immigration policy-making in Morocco and Tunisia given that the most obvious determinants - a change of political actors or a change in migration patterns - seem to not account for the observed immigration policy dynamics? The systematic investigation of this empirical paradox will be the central object of another article. The following sections present the core insights of this analysis, focusing on those that are essential for the theoretical arguments elaborated further below.

### Case study methodology

This article draws on my Ph.D. fieldwork in Morocco and Tunisia between October 2016 and May 2017, where I conducted 110 semi-structured interviews with (1) high-level civil servants directly involved in immigration policy-making (Ministry of Interior, Ministry of Foreign Affairs, Ministry of Migration) or key for the implementation of certain aspects of immigration policy (parliament, Ministries of Labor, Higher Education or Social Affairs, or local administrations); (2) representatives of civil society, be they migrant-led collectives, local NGOs working with migrants, labor unions, or international NGOs operating in Morocco and Tunisia; and (3) employees of international organizations such as of IOM, ILO, UNHCR and local EU delegations. Interviews were conducted in French and either recorded and transcribed, or followed through extensive note-taking. I also attended workshops and seminars on immigration policy during my stays in Rabat and Tunis to observe dynamics between actors and conduct another 50 informal conversations with additional respondents. Furthermore, I decided to integrate insights gained from 32 interviews conducted during a previous fieldwork in Morocco in 2011/2012 into the analysis.

Next to semi-structured interviewing, I did extensive online, library and archival research. I collected policy documents and institutional reports, minutes of parliamentary discussions, action plans and reports of local non-governmental organizations since the 1990s. I also systematically screened the official bulletins gathering laws and decrees in Morocco and Tunisia since 1956 and built a database on immigration policy change over the 20th and 21st century. The most challenging task was to gather Moroccan and Tunisian immigration statistics, given that they are often inexistent, fragmented, contradictory or scattered across sources.

The interviews and archival research provide the backbone of my analysis and were used to gather information on immigration policy developments and long-term transformations in Morocco and Tunisia; to establish a ‘cartography of the state’, i.e. the (changing) institutional and decision-making landscape sketching the power relations between different institutional, diplomatic, and civil society actors; and to ultimately reconstruct immigration policy-making. The next two sections outline the processes underlying the emergence of immigration as a ‘public problem’ in Morocco and Tunisia, as well as the way in which domestic and international institutions, interests, and ideas shaped immigration policy-making over the past two decades. The investigation of immigration policy-making in these two critical cases - Morocco’s stable monarchy as opposed to Tunisia’s ongoing democratization - provides the empirical foundation to investigate the broader role of political systems on immigration politics later in the paper.

### Morocco: Drivers and limits of immigration policy shifts

In 2003, Morocco passed its first immigration law since independence in 1956. Law 02–03 regulated foreigners’ entry and stay in Morocco, and criminalized irregular immigration and emigration. It also created the Direction of Migrations and Borders Surveillance within the Ministry of Interior, and led to the elaboration of a National Strategy on Combating Illegal Migration. The restrictive policy was overwhelmingly interpreted as a result of European pressure to control irregular migration. Yet, it first and foremost benefited Moroccan authorities’ domestic priorities and geopolitical standing (Natter, 2014b). Indeed, shifting the focus away from Moroccan irregular migrants and towards sub-Saharan ‘transit’ migrants appeased popular concerns, and provided Morocco with a new bargaining tool in their diplomatic relations with Europe. As a consequence, the 2003–2013 decade was characterized by an arbitrary, violent and security-driven state approach to immigration, forcing sub-Saharan migrants without legal status into miserable living conditions and constant fear of crackdowns or expulsion by Moroccan police forces (CMSM & GADEM, 2012).

In parallel, however, an active civil society started denouncing these practices by naming and shaming the Moroccan government on the international scene - at times very successfully. The role of CSOs over that period is particularly noteworthy, given that they operated in a political context of semi-authoritarian rule. The work of Caritas, the development of Moroccan associations such as the Anti-Racist Defense and Support Group of Foreigners and Migrants (GADEM), as well as of migrant organizations such as the Council of Sub-Saharan Migrants in Morocco (CMSM) has occurred in semi-legality. Interviews with representatives of these associations highlight that while they operated without legal status and were under constant police control, their activities were nonetheless tolerated (within certain limits) by the state. The Kingdom’s attempt to assert itself as a progressive, rights-respective country in the region might account for this hybrid approach towards CSOs.

Taking international and national observers by surprise, Moroccan King Mohamed VI announced the launch of a new, human-rights based immigration policy on 10 September 2013. Its centerpiece was a regularization campaign, a premiere in the North African context, that granted legal status to nearly 26,000 irregular migrants, mainly Syrian and sub-Saharan refugees, European and sub-Saharan workers and family members living in Morocco for years (CNDH, 2015). The newly created Moroccan Ministry for Moroccans Residing Abroad and Migration Affairs (MCMREAM) was tasked with implementing the National Strategy on Immigration and Asylum (SNIA), elaborating laws on asylum, human trafficking and immigration, and overseeing integration measures in the health, labor and education sectors. In December 2016, a second regularization campaign was launched.

The motives underlying this shift have left many puzzled, but the configuration of international priorities, national political developments, and specific institutional interactions provides some explanations. First, the new policy can be cast as a change in ‘Moroccan geopolitical culture’ (Cherti & Collyer, 2015), partly away from Europe and more directed towards Africa. Indeed, since 2010, King Mohamed VI has embarked on a pro-active Africa policy, characterized by multiple diplomatic visits, the signature of heavy trade deals and efforts to rejoin the African Union (AU) - which Morocco left in 1987 and successfully rejoined in January 2017 (Rousselet, 2015). Morocco’s interests in Africa were thus increasingly at odds with the violence and maltreatment of African nationals by Moroccan police forces and border guards. Also, the denial of immigrants’ rights in Morocco stood in stark contrast to Morocco’s lobbying for more migrants’ rights in Europe.

Yet, the new migration policy not only reflects a change in Morocco’s foreign policy strategy, but also emerged out of national political developments. Most importantly, the 2011 constitution created the National Council of Human Rights (CNDH), an independent state body headed by Driss El Yazami, a former political refugee in France and human rights activist. Its activities, notably the September 2013 report on migrants’ rights abuses perpetrated by the Moroccan state, had wide-ranging consequences for the treatment of migration in Morocco, as it eventually triggered the launch of the new migration policy.

A few years down the line, the policy is in implementation. Refugees and regularized migrants have obtained stay permits, eased access to the labor market, education and public health care. Other changes are underway to facilitate the integration and social acceptance of immigrants. Yet, the sustainability of the new approach remains uncertain in the face of continued raids of irregular migrants’ settlements and migrants’ rights violations, especially in the North of Morocco and in the border areas (FIDH/GADEM, 2015). One of the reasons for these territorial inconsistencies is institutional: While the Migration Ministry is overseeing the new migration policy, its successful implementation depends on sectoral ministries such as the Ministry of Health, Education, Interior or Labor, for whom migration is not a priority. Thus, despite the discursive adherence of all institutions to the new policy, it has been a challenge for the Migration Ministry to put immigration on these ministries’ agendas.

Also, the new policy has led to a mushrooming of migration-specific CSOs in Morocco that act as intermediaries between migrants and the state. This development has created issues of competition, cooptation, and legitimacy. It has also fragmented civil society, affecting its capacity to provide a robust counter-discourse to official discourse. Interviewees, however, highlighted that the main challenge is to integrate ad-hoc policy decisions into national legislation in order to protect the new policy from future backlashes. As long as the laws on immigration and asylum, finalized but stuck at the political level, are not enacted, Morocco’s immigration policy will thus remain ambivalent and arbitrary (Norman, 2016a).

### Tunisia: Democratization as opportunity and challenge

In Tunisia, the two main laws regulating immigration today date back to 1968 and 1975 and are waiting to be reformed. Reacting to growing European demands to control ‘transit’ migration, Ben Ali’s authoritarian regime developed a two-pronged immigration policy that has led to the generic silencing - or ‘omertà’ - of immigration in the public sphere. While sub-Saharan students and employees of the African Development Bank were welcomed by the regime as ‘guests’ and received state protection from eventual racist assaults, irregular immigration was managed solely through the security lens. In 2004, Tunisia restricted existing sanctions for irregular migration through law 04–06. The law not only pleased European partners but was also a state survival tool, as it allowed Ben Ali’s regime to better monitor the movements of both citizens and foreigners on Tunisian territory (Boubakri, 2009). While the law has been widely denounced for its human rights violations, the lack of an active civil society until 2011 has limited the effectiveness of those criticisms (Beau & Tuquoi, 2011; Camau & Geisser, 2003).

From the outset, revolution and migration were intrinsically linked. Within the first few months of 2011, the absence of Tunisian border controls resulted in a temporary hike of irregular emigration of Tunisians towards Italy and the much larger and prolonged arrivals of refugees, labor migrants and Libyan citizens from Libya (see Boubakri, 2013; Natter, 2015). The political events of 2011 set migration on the public and political agenda - triggering two contradictory trends. On the one hand, the unprecedented increase in civil liberties prompted significant civil-society activism. Existing CSOs embraced migrants’ rights in their work, such as the Tunisian Forum for Economic and Social Rights (FTDES) or the Tunisian Association of Sub-Saharan Students (AESAT), and new CSOs were created to protect irregular migrants and workers, such as Terre d’Asile Tunisie (TAT). On the other hand, the liberalization of the public sphere also led to racist backlashes against migrants. While protected by Ben Ali’s regime, sub-Saharan migrants report a growth of verbal and physical racist attacks after 2011, as their presence in Tunisia was intimately related to the old regime. These particularly complex interlinkages between migration and politics have hindered the solidarization between Tunisians and migrants in the post-revolutionary period.

Two other important dynamics played in favor of politicizing migration in the immediate post-revolutionary period. First, the integration into Tunisian institutions of numerous leftist and Islamist political figures who had returned from exile explains the initial dynamism on migration-related issues. A new State Secretariat of Immigration and Tunisians Abroad (SEITE) was created in 2012 to develop a national migration strategy (dormant since 2015 and still not officially adopted) and the Ministry of Justice started elaborating two laws - a law against human trafficking passed in August 2016, and a law on asylum currently blocked at the political level. Second, the multitude of international organizations that arrived in Tunisia brought not only funds, but also their interests and discursive frameworks with them. IOM and UNHCR, the German, Swiss and French Development cooperation or groups such as Euromed Rights have - relatively successfully - increased the pressure on Tunisian state institutions to deal with immigration in a process that could be coined ‘external agenda setting’: Although Tunisian authorities still consider Tunisia mainly as a country of emigration and transit, a discursive change is visible, as a growing number of public officials start to incorporate the destination country dimension into their discourse.

Yet, the democratization has not led to the expected major revision of immigration policy. After initial dynamism on migration-related issues between 2011 and 2014, migration was put on the backburner and efforts to get rid of the security focus have remained unsuccessful. How can we understand the absence of a real policy change? First of all, governmental volatility has considerably slowed-down decision-making in all public policy areas, and the fragmentation and disempowering of the administration under Ben Ali has left serious marks on inter-institutional coordination and trust. The SEITE has particularly suffered from this: First created in 2012 within the Ministry of Social Affairs, it was dissolved and recreated multiple times, most recently in August 2016 under the umbrella of the Ministry of Foreign Affairs. The ensuing turf war over the migration dossier between the Ministries of Social and Foreign Affairs has hardened the stalemate around immigration, partly resolved in September 2017 through the return of the SEITE to the Ministry of Social Affairs.

Geopolitical considerations can also partly explain the absence of immigration policy change - especially towards the considerable number of Libyan citizens in Tunisia. With two Libyan governments next door, Tunisia is wary of taking sides in the internal Libyan conflict to secure future cooperation and trade with Libya no matter the outcome of the domestic struggle. The non-treatment of the administrative situation of Libyan citizens in Tunisia reflects this strategic neutrality towards Libya: they are neither considered migrants, nor refugees, but ‘guests’ or ‘brothers’. This laissez-faire approach was viable at first, but the continued presence of several hundreds of thousand Libyans in Tunisia starts to raise practical issues of access to property, public healthcare, and education.

While these determinants for policy continuity are likely to endure, Tunisia’s National Assembly could become a driver for more protective laws on immigration. In fact, a draft law criminalizing racist behavior was submitted to parliament by a Tunisian NGO in summer 2016 and has received support from a substantial number of MPs. In January 2018, a revised version of the law has been adopted by the Council of Ministers and is awaiting adoption by the parliament. Also, with 18 MPs elected by Tunisian citizens living abroad, Tunisian emigrants could feed some of their migratory experiences back into the Tunisian parliamentary discussions, creating what one might call ‘political remittances’ (Lacroix, Levitt, & Vari-Lavoisier, 2016; Levitt, 1998).

## Rethinking theory

### Bridging political sociology, public policy, and migration research

Before confronting my empirical analysis with existing immigration policy theories, I would like to highlight three insights from political sociology and public policy research that are key for the theorization of immigration policy-making regardless of the political system in place. **First**, the state is not a uniform, rational actor, but consists of fragmented institutions that can pursue multiple, potentially contradicting goals. ‘Autocratic’ policy-making is often wrongly assumed to be so centralized as to not leave any room for contestation, negotiation, and lobbying. Although executive powers usually have higher decision-making leverage in autocracies compared to liberal democracies, where democratic oversight and power balances create an inbuilt need for compromise, this overlooks the fact that non-democratic regimes also need to sustain their legitimacy and survival (Brooker, 2014; Bueno de Mesquita, Smith, Siverson, & Morrow, 2003). They too negotiate decisions with economic and political actors within and outside the state apparatus - dynamics that are worth investigating to understand immigration policy-making in those contexts. In Morocco, the King’s power explains the extraordinary dynamism on immigration since 2013, and the discursive adherence of different state institutions (most notably the Ministry of Interior) to the new policy. Yet, while this has avoided open disagreement, the royal will did not preclude power politics between different state institutions.

**Second**, policy-making in modern states is usually characterized by incoherencies and discrepancies between policy discourses, policies on paper and policy implementation (Czaika & de Haas, 2013). Enacted policies are often watered-down versions of originally-stated policy intentions because the decision-making process requires the reconciliation of different interests. This has been widely documented for ‘Western’ immigration policy-making (Boswell, 2007; Joppke, 1998), but there is no reason to restrict this insight to liberal democracies. In Morocco for instance, interviewees have attested the striking territorial differences in the implementation of the new migration policy, whereby the situation of migrants in Moroccan cities (Casablanca, Rabat, Fez) is much better than in places such as Oujda, the northeast or on the countryside - be it regarding their access to social services or the renewal of their legal stay permit.

**Third**, public policy theories have emphasized the crucial role played by individuals in policy-making, be they politicians, bureaucrats or civil society activists. While this aspect has not yet gained much attention in immigration policy theory, it is an area worth investigating. In Morocco and Tunisia, interviews have shown the critical role played by particular individuals in triggering policy change, many of whom were previously emigrants themselves. These ‘political remittances’ were fed back into national institutions through the integration of previously exiled individuals into the political system, such as the current president of the National Council of Human Rights (CNDH), Driss El Yazami, in Morocco or leftist and Islamist political figures who entered Tunisian political institutions after 2011. For instance, the rapidity with which Tunisia concluded the UNHCR headquarter agreement in 2011 might have been facilitated by the fact that the State Secretary of the Tunisian Minister for Foreign Affairs between January and December 2011 had been the head of UNHCR for the Middle East and North Africa region until 2010.

Integrating insights from political sociology and public policy into migration studies, and acknowledging some fundamental similarities across different political systems is a first necessary step to improve immigration policy-making analyses around the world. The second step is the systematic confrontation and dialogue between new empirical material and existing theories to challenge often implicit assumptions about the nature of immigration policy-making. The following sections seek to disentangle those theoretical explanations that provide generic insights into immigration policy processes from those that portray a ‘regime effect’.

### Issue-specific immigration policy processes

Four of the theoretical lenses outlined in the introduction provide insights into immigration policy-making that seem valid regardless of the political system in place, as they capture dynamics inherent to the issue of immigration. **First of all**, the institutionalist literature with its emphasis on inter-institutional turf wars and bureaucratic politics provides a relevant theoretical framework to look at immigration policy-making across different political systems. For instance, institutions with different worldviews are likely to adopt different positions on immigration. Ministries of Interior will mostly follow a security-driven agenda, privileging an approach that maximizes control over human mobility, while Ministries of Foreign Affairs will be tempted to use migration as a diplomatic tool - ready to sacrifice policy coherence over time if required by circumstances -, and Ministries of Health will be more sympathetic to opening services to foreigners given the imperative of securing public health. These diverging visions on immigration can initiate turf wars that are familiar from countries such as France, Germany or Spain.

Inter-institutional incoherence is, however, equally visible in Moroccan and Tunisian immigration policy-making. In Tunisia, sub-Saharan African students face partly incompatible rules from different ministries that make their timely regularization almost impossible. While the application for a stay permit at the Ministry of Interior requires a proof of enrollment by the university, the Ministry of Higher Education allows universities to issue these proofs only one month after classes start, while the Ministry of Finance issues financial sanctions for every week spent irregularly in Tunisia. The Kafkaesque nature of these administrative requirements is by no means specific to Tunisia, but they highlight the importance of paying attention to inter-institutional dynamics.

**Second**, the role of global policy diffusion and international norms in shaping national immigration policies is not limited to ‘Western liberal democracies’. (Nearly) all countries seek to cooperate with other global and regional powers to secure political and economic survival for their rule at home (Cassarino, 2014; Escribà-Folch & Wright, 2015; Risse, Ropp, & Sikkink, 1999). In fact, in his initial formulation of the embedded liberalism hypothesis, Hollifield (1992b, p. 578) mentioned that these processes could also be relevant in less liberal contexts: “Respect for human (and civil) rights can compel liberal states (and some that are not so liberal […]) to exercise caution in dealing with migrants.” Morocco, for instance, signed the ILO convention on migrant workers’ rights in 1991, when it was still a prototypical origin country, hoping it would substantiate its call for more rights to Moroccan migrants in Europe. Two decades later, Moroccan CSOs use this convention to underpin their call for more migrants’ rights in Morocco, drawing on Morocco’s commitment to a global standard of rights.

However, while democracies are exposed to international norms through hard instruments such as laws, conventions, and courts; liberal norm adherence might play out more strongly in autocratic systems that seek to portray themselves as progressive countries on the international scene. As stated by FitzGerald and Cook-Martín (2014, p. 21): “One of the purposes of immigration and emigration policies is to make a country appear more modern and civilized. Migration policies are dramaturgical acts aimed at national and world audiences.” The recent adoption of laws against human trafficking in both Morocco and Tunisia exemplify these dynamics. While the positive effects of such legislation on the situation of victims of human trafficking remain uncertain, these laws signal international partners a symbolic adherence to a global epistemic community and system of norms.

**Third**, the importance of foreign policy considerations in immigration policy-making (Boswell, 2003; Cassarino, 2014; Mitchell, 1989; Teitelbaum, 1984) is not limited to ‘Western liberal democracies’. In fact, in countries simultaneously experiencing immigration, ‘transit’ migration and emigration, the international dimension of migration politics might be particularly important. Inspired by the concept of the ‘two-level game’ (Putnam, 1988) that captures parallel decision-making on domestic and international political spheres, these countries seem exposed to a more complex ‘three-level game’, where diplomatic interests towards countries of origin and destination compete alongside domestic interests. With this in mind, Morocco’s regularization campaigns in 2014 and 2017 can be understood either as a rebalancing of geopolitical interests towards Africa or as a ‘demonstration effect’ towards Europe. In the latter interpretation, Morocco’s liberal immigration policies form an integral part of its emigration policy strategy, seeking to demonstrate to European host countries that a more forthcoming treatment of immigrants is possible. This policy linkage strategy is particularly tempting when immigration is relatively low.

The presence of a three-level game indicates a potentially stronger weight of foreign policy considerations in immigration policy compared to domestic concerns because the ‘public’ for which immigration policies are made is not necessarily the electorate (the local population needed to sustain the legitimacy of the government), but international cooperation partners required for regime survival. In fact, foreign policy priorities can partly explain the empirical puzzle introduced earlier regarding the disconnection between the political salience of migration and its numerical magnitude. While the Moroccan state has decided to instrumentalize and inflate sub-Saharan migration for its African and European diplomacy, the Tunisian state has deliberately ignored Libyan immigration to escape its politicization - in both cases a political decision detached from the actual size of the phenomenon.

**Finally**, the premises of the national identity approach are highly valuable to analyze the origins and drivers of immigration policy-making regardless of a country’s political system. As has been famously stated by Sayad (1999, p. 6) – “penser l’immigration, c’est penser l’Etat” – thinking about immigration means thinking about the state. Recently, Klotz (2015) showed how current South African immigration policy is rooted in a century of state formation and societal discussions over national identity. In Tunisia, interviewees emphasized the difficulty of politicizing racism (be it against sub-Saharan migrants or black Tunisians) in a post-revolutionary context where political leaders continuously highlight the unity of Tunisian national identity. In Morocco, immigration policy has been used as a tool for ‘political rebordering’, as the new migration agenda provided an unequaled opportunity to showcase the liberal and progressive character of the Moroccan state, and to cast itself as a ‘global migrant destination’ and thus part of the ‘developed world’.

### Regime-specific immigration policy processes

This paper also identified three immigration policy theories that are prone to a regime effect, as they rely on essential features of the liberal democracy that are absent in more authoritarian regimes. **First of all**, given the fundamentally different role of public opinion, mass media, the electorate and political parties, the domestic politics approach is not - at first sight - easily applicable to more autocratic political systems that seem, indeed, exposed to less influential domestic actors. The Tunisian case shows this regime effect, as the democratic transition has transformed civil society from a small group of threatened activists into a dynamic and powerful, although sometimes unstructured and fragmented, voice on the political scene. For Tunisian CSOs working on migration, migrants’ rights have become a yardstick to assess the success or failure of the ongoing democratization.

**Second**, and relatedly, Freeman’s client politics hypothesis does not neatly apply to regimes that do not have to balance electoral considerations with pressure from organized interest groups. Yet, domestic politics approaches and, more specifically, Freeman’s costs and benefits rationale should not be disregarded as tools to understand policy-making processes in more autocratic systems. In contrast to liberal-democratic regimes whose survival is bound to public support and materialized in electoral victories and parliamentary majorities, autocratic regimes assure their survival through repression or international support, but also through the cooptation of specific segments of the population (Brooker, 2014). In this vein, Bueno de Mesquita et al. (2003) have argued that authoritarian states rely not on an ‘electorate’, but on a ‘selectorate’ for their survival. The main questions should, therefore, be: Who is the ‘selectorate’, who are the clients of these countries’ immigration policies?

Among the ‘clients’ highlighted by existing research are interest groups such as labor unions and business lobbies, as well as CSOs - actors that are often institutionalized and explicitly part of the policy process in liberal democracies. While previous research has shown the power of civil society even in more autocratic settings (Natter, 2014b; Russell, 1989), the influence of CSOs might be either more indirect or more informal. On the one hand, local CSOs might influence policy processes only through international support (see Keck & Sikkink, 1992). This external pressure can, in turn, challenge the legitimacy of the regime and influence policy decisions. On the other hand, CSOs might play an even larger role in autocratic than in democratic systems, precisely because formal institutions do not effectively channel popular demands into policy processes. Morocco is a good example for both processes: local CSOs have been successfully denouncing Morocco’s migrants’ rights abuses in cooperation with international actors since the early 2000s, and since 2013 CSOs have become the privileged partner for authorities to implement the new migration agenda, side-lining political parties who have not seized the immigration issue for their political agendas so far.

**Third**, the role of epistemic communities of lawyers and judges in safeguarding and expanding migrant’s rights (Joppke, 1998) is prone to a regime effect, as lawyers and courts cannot act as brakes for immigration restrictions in regimes with a weak rule of law. This does not mean that there is no legal action on migration in more autocratic regimes, but their influence will be limited. In Morocco, for instance, lawyers have started challenging inconsistent and arbitrary court decisions on certain immigration-related issues over the past years. While this bottom-up approach has created a small jurisprudence on immigration that could set precedents, it has not played a crucial role in triggering policy reform. In Tunisia, interviews with CSOs, lawyers, and judges attest an increasing awareness and disposition to use the rule of law and court judgments to secure immigrants’ rights in the future. Accordingly, it seems a question of time, financial support, and professionalization of Tunisian CSOs until the democratic transition will also materialize in establishing courts as defenders of migrants’ rights.

**Finally**, inspired by Hollifield’s ‘liberal paradox’ and the Moroccan case study, this paper seeks to advance the hypothesis of an ‘illiberal paradox’. Hollifield posits that the dominant ideology of liberalism has pushed (labor) markets to globalize and has enshrined international human rights into national immigration legislation, while the political logic of liberal-democratic nation-states, dominated by electoral objectives, is one of closure and immigration restrictions. This leads to a situation in which discourses about immigration are often more restrictive than policies in practice. In contrast, the ‘illiberal paradox’ hypothesis posits that autocratic, illiberal regimes, while bound by the same international forces of liberalism, are more autonomous from nation-state logics asking for closure and can thus more easily enact liberal immigration policies if it fits their priorities. For instance, autocratic or semi-autocratic regimes have enacted surprisingly liberal immigration policies, based on their economic or foreign policy interests - such as Morocco’s monarchy since 2013, but also Ghaddafi’s regime in the 1990s or Uganda since 2006.

Relatively independent from potential popular anti-immigration sentiment, and not bound by existing legal frameworks and path dependency to the same extent as consolidated democracies, fundamental policy shifts are easier to enact. While this can favor more liberal migration policies, it also increases the vulnerability of these liberal policies and the risk of a sudden backlash - as has been observed, for instance, in Libya after 2000. More generally, the ‘illiberal paradox’ hypothesis might be particularly relevant for countries where immigration remains numerically rather low, or where liberal policies can be contained to specific aspects and do not spill over into a general liberalization of immigration and immigrant integration. A final limitation is that this discursive openness towards immigration is not always followed-through in practice because autocratic states are often prone to corruption, the weak rule of law throughout the territory and an arbitrary implementation of policies. Thus, discourses about immigration are often more liberal than enacted policies - creating an exact opposite situation as the one described by Hollifield and his liberal paradox.

## Conclusion

This paper aimed to rethink immigration policy theories beyond so-called ‘Western liberal democracies’ with three interrelated ambitions in mind. First, to go beyond the simplistic and often overrated dichotomy between autocracy and democracy in immigration policy analysis and, instead, adopt an approach focused on the structure, functioning and practices of a country’s political system. Second, to abandon homogenizing understandings of the state and to recognize its internal fragmentations - regardless of the political system in place. Third, to take existing immigration policy theories not as mutually exclusive explanations, but to see to what extent they can be combined, consolidated or adapted to the empirical material at hand - following a theory-building rather than theory-testing approach. This comes with the trade-off of not providing a single ‘meta-theory’ of immigration policy that is testable in a positivistic sense. To fully realize the potential of the comprehensive approach advocated in this paper, in-depth, detailed case studies should investigate how changing configurations of explanatory factors shape immigration policy-making.

Starting from the empirical puzzle on the dislocation between the magnitude of immigration, the politicization of immigration and political developments in twenty-first century Morocco and Tunisia, this paper suggested a two-dimensional categorization of immigration policy processes. On the one hand, some insights on immigration policy-making theorized by the literature on ‘Western liberal democracies’ are in fact present across a variety of political systems, as they are intrinsically linked to the issue of immigration: (i) Bureaucratic politics analyses focusing on institutional turf battles and the diverging worldviews of state actors are crucial to understand policy developments regardless of the political system in place. (ii) Autocratic regimes are also exposed to the constraints and diffusion of liberal norms, although they are more likely to exert an indirect pressure on the country’s symbolic politics rather than act through legal instruments. (iii) The weight of diplomatic and foreign policy considerations might be even higher in non-democratic regimes due to the presence of a ‘three-level-game’ in immigration policy-making, in which decision-makers have to juggle with domestic interests and diplomatic interests towards origin and destination countries, eventually linking immigration and emigration politics along the way. (iv) Finally, the national identity approach allows understanding the fundamental vectors that structure a country’s approach to immigration regardless of the political system in place.

On the other hand, the paper has advanced the ‘illiberal paradox’ hypothesis to account for the enactment of liberal immigration policies by rather illiberal, autocratic states. Three features of immigration policy-making that are indeed prone to a regime effect can account for the fact that, in reaction to economic or diplomatic priorities, autocratic regimes have more leverage to enact liberal immigration policies in relative autonomy from potential societal demands for restriction: (i) Most notably, the weight of the electorate and political parties - highlighted by the domestic politics approach - is by definition much lower in more autocratic systems, and reduces the pressure of popular demands on decision-makers. (ii) While not blindly applicable to more autocratic regimes, Freeman’s client politics theory however invites a reflection on the role of the ‘selectorate’ in autocratic regimes, and a discussion about who the ‘clients’ of these countries’ immigration policies are. Next to segments of the political and economic elites, CSOs can influence autocratic decision-making processes decisively, even if their role is expected to be either more indirect, passing through external support, or more central than in liberal democracies, acting as a replacement for formal democratic processes. (iii) Finally, in political systems where the rule of law is weak, the role of independent lawyers and courts as a counterweight to executive or legislative policy decisions appears to be significantly limited, sometimes even inexistent.

As part of my Ph.D., the hypotheses developed in this paper are by no means final claims. Instead, they will hopefully serve as starting points for further discussions about and refinement of migration policy theory. Given that one out of two international migrants live in countries that have been omitted by immigration policy theories so far, I believe it is paramount to analyze and bring to the fore the immigration histories and policy processes in other countries in the world to achieve a more genuine and global understanding of the role of states and their policies in international migration.
